# Technical Feasibility and Design of a Shape Memory Alloy Support Device to Increase Ejection Fraction in Patients with Heart Failure

**DOI:** 10.1007/s13239-018-00399-7

**Published:** 2019-01-09

**Authors:** K. M. Aarnink, F. R. Halfwerk, S. A. M. Said, J. G. Grandjean, J. M. J. Paulusse

**Affiliations:** 1 University of Twente, TechMed Centre, Enschede, The Netherlands; 2 Isala Hospital, Zwolle, The Netherlands; 3 Department of Cardio-Thoracic Surgery, Thorax Center Twente, Medisch Spectrum Twente Hospital, PO Box 50 000, 7500 KA Enschede, The Netherlands; 4 Department of Biomechanical Engineering, TechMed Centre, University of Twente, Enschede, The Netherlands; 5 Department of Cardiology, Hospital Group Twente, Almelo-Hengelo, The Netherlands; 6 Department of Biomolecular Nanotechnology, University of Twente, Enschede, The Netherlands; 7 Department of Nuclear Medicine and Molecular Imaging, University Medical Center Groningen, Groningen, The Netherlands

**Keywords:** Shape memory alloy, Heart failure, Ejection fraction, Cardiac support device, Design configuration

## Abstract

**Purpose:**

Heart failure is increasingly prevalent in the elderly. Treatment of patients with heart failure aims at improving their clinical condition, quality of life, prevent hospital (re)admissions and reduce mortality. Unfortunately, only a select group of heart failure patients with reduced ejection fraction are eligible for Cardiac Resynchronization Therapy where 30–40% remain non-responders and need left ventricular support. The aim of this study is to investigate if a shape memory alloy (SMA) is able to increase the ejection fraction of a mono-chamber static heart model by 5%.

**Methods:**

A pediatric ventilation balloon was used as a heart model (mono-chamber). Flexinol^®^, a SMA, was placed around the heart model in multiple configurations and activated using pulse width modulation techniques to determine influence of diameter and configuration on volume displacement. Furthermore, pressure within the heart model was measured with a custom-made pressure sensor.

**Results:**

SMA with a diameter of 0.38 mm, placed in a spiral shape and activated with a duty cycle of 80% and a frequency of 50/min gave the highest ejection fraction increase of 3.5%.

**Conclusions:**

This study demonstrated the feasibility of volume displacement in a static heart model by activation of SMA-wires. Configuration, duty cycle, frequency, pulse intervals and diameter were identified as important factors affecting the activation of SMA-wires on volume displacement. Future research should include the use of parallel SMA-wires, prototype testing in dynamic or *ex vivo* bench models.

**Electronic supplementary material:**

The online version of this article (10.1007/s13239-018-00399-7) contains supplementary material, which is available to authorized users.

## Introduction

### Epidemiology and Current Practice

Heart failure (HF) is characterized by the “inability of the heart to pump blood at an adequate volume”^26^ and results in marked limitation of physical activity. Over 6.5 million Americans have HF and increases by 46% in 2030.^3^ HF is an incurable, lethal disease, where 1 in 8 deaths are related to HF with a 5-year mortality rate after diagnosis of HF of 50%.^8^ Also, HF is the most expensive diagnosis for hospitalizations and 30-day readmissions.^6^

Treatment of heart failure focuses on quality of life improvement, prevention of hospital admissions and aim to decrease mortality. For patients with a reduced left ventricular ejection fraction (HFrEF) below 40%, pharmacological treatment with angiotensin-converting enzyme inhibitors, beta-blockers and mineralocorticoid/aldosterone receptor antagonists improve survival rates.^19^ To reduce the risk of sudden death, implantation of an Implantable Cardioverter Defibrillator is recommended. For symptomatic patients, Cardiac Resynchronization Therapy (CRT) is indicated for a select group of patients, yet over 30% of patients are non-responders and do not benefit from this treatment.^2^ For patients with resistant symptoms, surgical implantation of a Left Ventricular Assist Device (LVAD) or heart transplantation may be considered,^19^ despite drawbacks of infection of the external battery driveline occurring up to 35%^14^,^16^ and an increased risk for thrombogenicity and thrombosis.^1^

### Technological Opportunities

Shape Memory Alloys (SMAs) are able to recover their shape after unloading from a particular stimulus, such as temperature.^9^ At high temperature, these SMAs contract and reverts to its original state during the cooling process.^11^ Application of SMAs in medical practice, such as coronary stents, has continued to emerge since its discovery in the early 1930s.^17^ SMAs have effective strain generation up to 4%, with fatigue between 4 and 8%, high strength to weight ratio, and low operating voltage.^15^,^20^ Recently, the use of SMAs in the treatment of heart failure in bench models generated considerable attention.^12^,^23^ A mechano-electric artificial myocardial assist system using SMA-wires augmented aortic flow rate by 15% in a goat study. Also, systolic left ventricular pressure was elevated by 7% under the cardiac output condition of 3L/min in these goats.^21^

However, challenges remain with respect to energy supply, relaxation times of SMAs and testing in realistic heart models.

In this demonstrator study, we investigate which SMA designs, and which Pulse Width Modification (PWM) techniques are able to increase ejection fraction of a mono-chamber static heart model by at least 5%.

## Materials and Methods

### Materials

**Figure 1:**
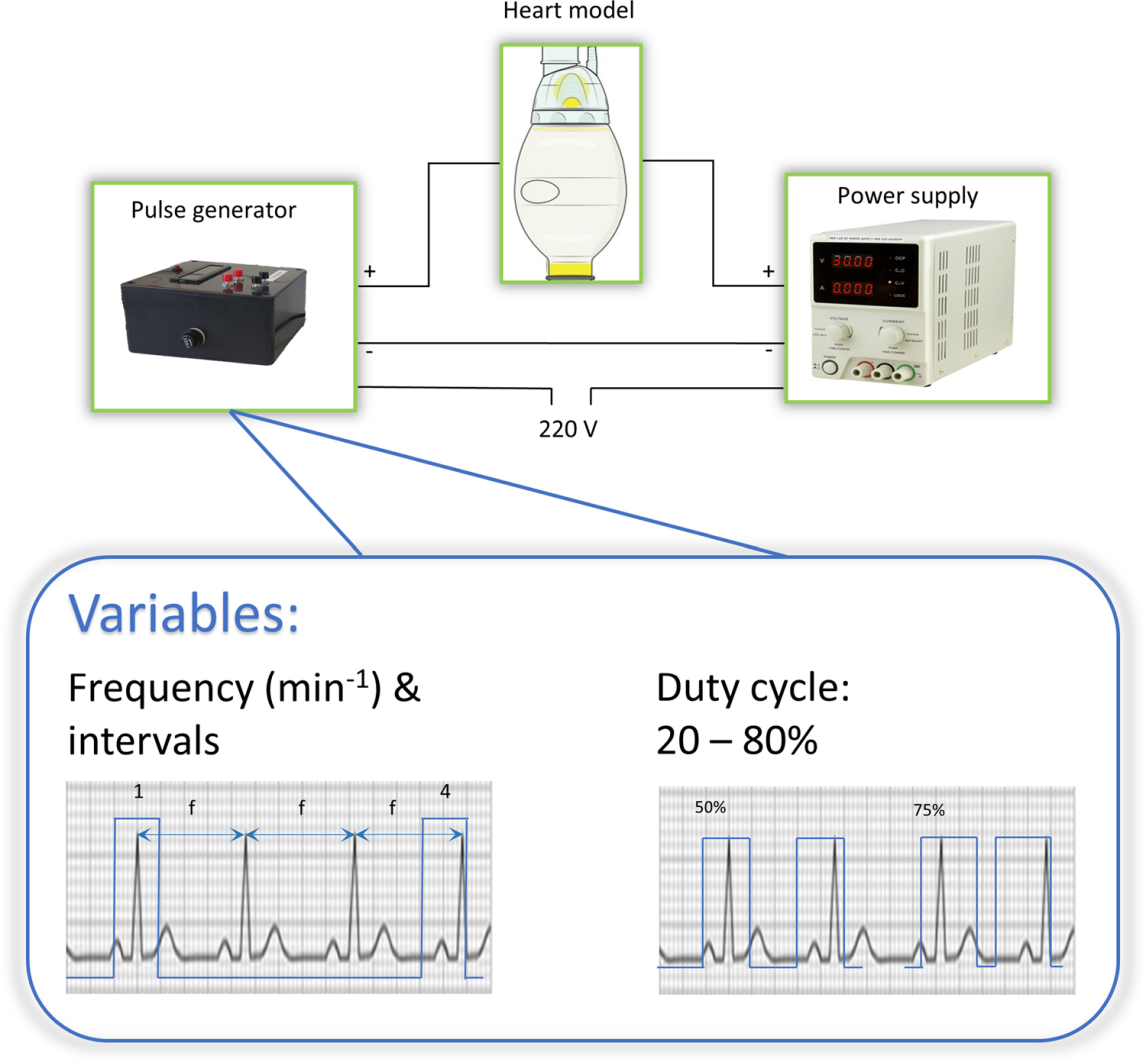
Schematic representation of test setup. Pulse generator which activates the SMA around the heart model with three major variables: frequency, duty cycle and pulse intervals.

**Figure 2:**
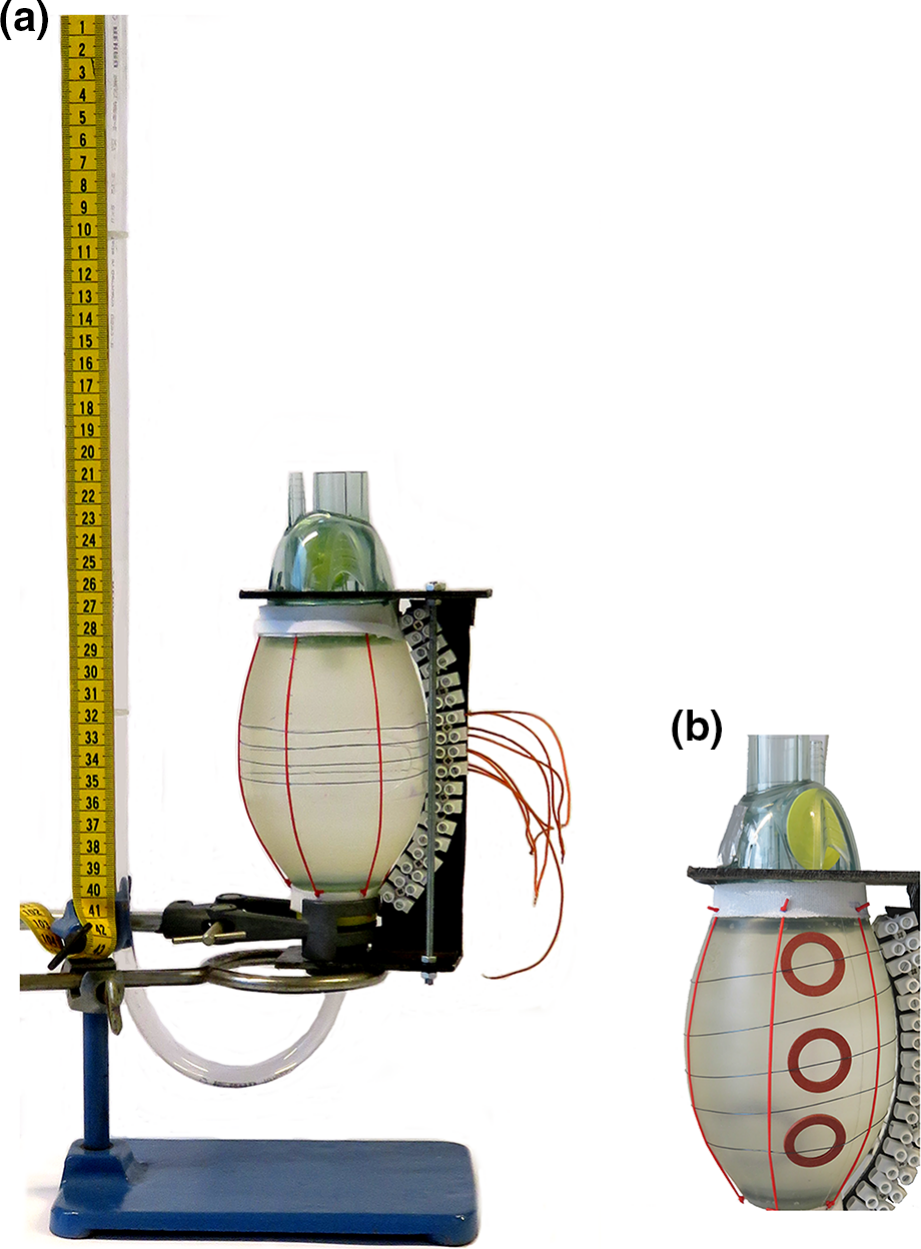
(a) Test setup with ventilation balloon as a heart model; (b) detailed overview of heart model.

A pediatric silicone resuscitation balloon (Laerdal Benelux bv, Amersfoort, The Netherlands) was used as a mono-chamber static model with a volume of 475 mL. Flexinol^®^ actuator wires (Dynalloy Inc., Irvine, California, United States) were used as SMA. Flexinol^®^ wires are composed of nickel-titanium alloy (55_wt%_Ni, 45_wt%_Ti) with a diameter of 150, 250, and 380 *μ*m respectively, displaying transformation temperatures of 70 °C. The density of Flexinol^®^ is 6.45 g/cm^3^ with a heat capacity of 0.2 cal/g K and thermal conductivity of 0.18 W/cm K.^4^,^5^ A detailed description of nickel-titanium alloys can be found elsewhere.^12^ A power supply and pulse generator were used to measure volume displacement after activation of SMAs (Fig. 1). A skeleton composed of Flexinol^®^ wires, micro-tubes, vascular ties, rings and Velcro straps was used based on the experimental setup of Shirashi *et al*.^15^ and is depicted in Fig. 2a. Rings were positioned to distribute force more evenly over the balloon. SMA wires were connected to small power cables by cable shoe connectors and attached to screw terminals for proper fixation (Fig. 2b).

### Design Configurations

**Figure 3:**
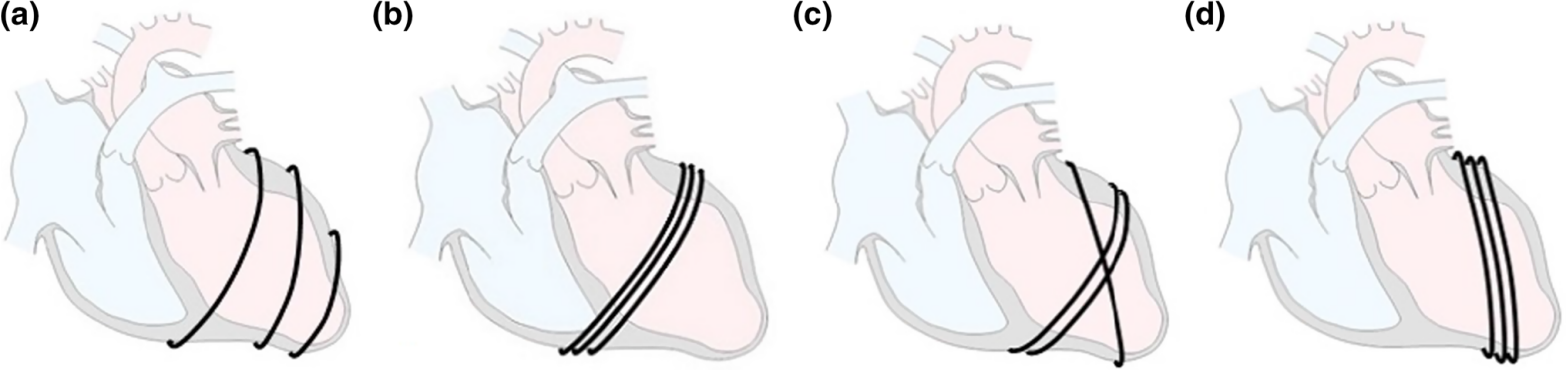
Conceptual artwork of configurations with (a) spiral; (b) band; (c) cross; (d) oblique orientations.

Different configurations of SMA-orientation were evaluated, e.g. spiral, band, cross and oblique shaped orientations (Fig. 3) at a frequency of 20/min. Volume displacement was measured in a transparent open top plastic tube (height 400 mm, diameter 8 mm), vertically connected to the resuscitation balloon. Both balloon and part of the vertical plastic tube were filled with water (Fig. 2a). First two pulses were used to warm-up the SMA-wire and stabilize the system.

Images were recorded with a compact camera, processed by dedicated software using Kinovea 0.8.15 (France) where volume level was tracked as a function of time for all design configurations. Displaced volumes and corresponding ejection fractions were calculated using Microsoft Excel 2016. Graphs were made with GraphPad Prism 5 (GraphPad Software, Inc., San Diego, CA, U.S.A.).

PWM techniques were used (Fig. 1) to alter pulse frequency (50–90/min), duty cycle (20–80%) and pulse intervals (1–5) to elicit maximal volume displacement of a single SMA-wire.

### Pressure Measurements

**Figure 4:**
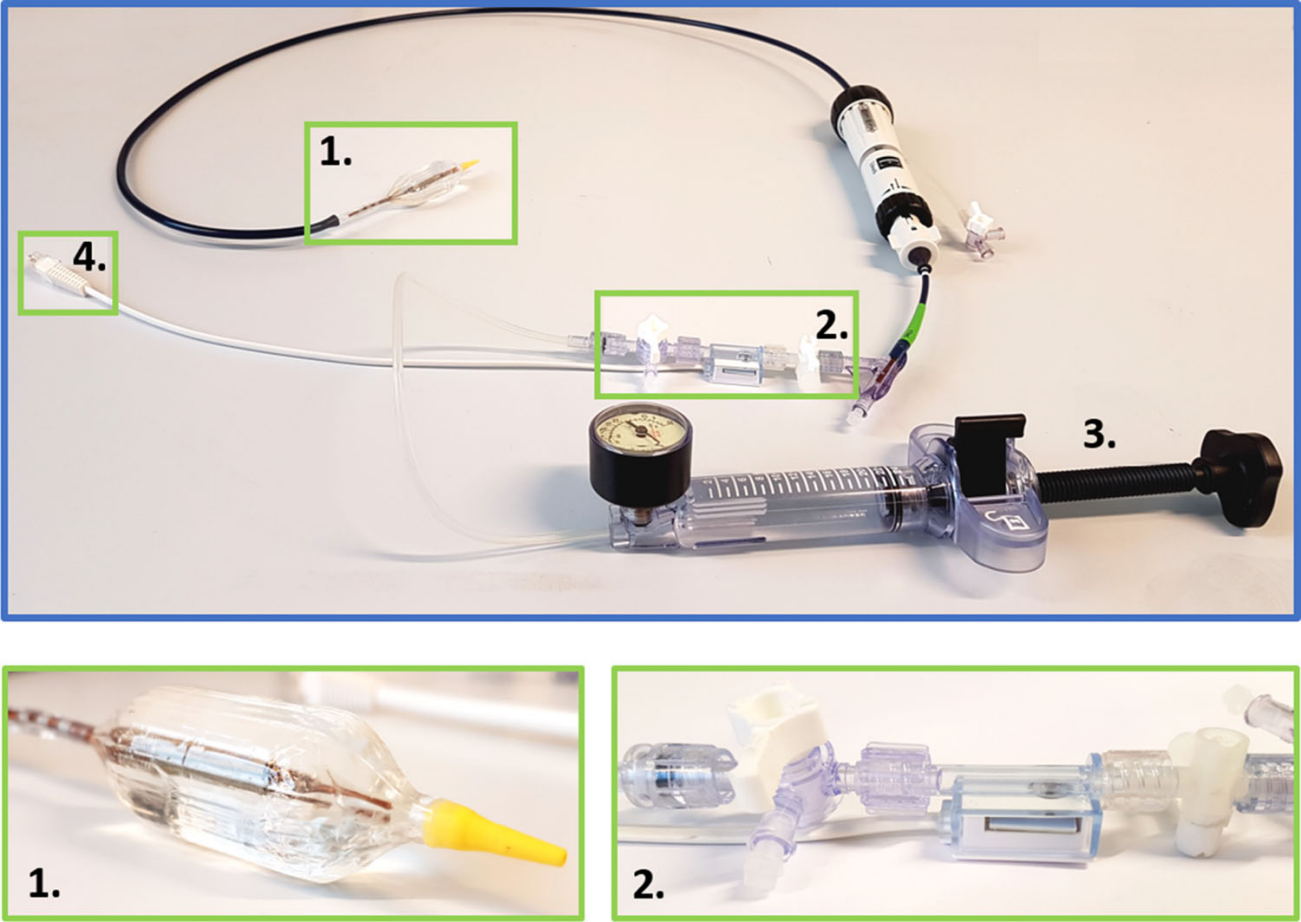
Pressure sensor. (1) inflated Transcatheter Aortic Valve Implantation (TAVI) balloon; (2) external pressure sensor; (3) manual pump with pressure gauge to fill TAVI-balloon with water; (4) connection to pressure sensor and microcontroller.

Pressure development was assessed within the resuscitation balloon according to ∆*P* =* p *×* g *×* h*: where *p* is density of the liquid kg/m^3^, *g* is gravitational acceleration m/s^2^ and *h* is the height difference of the liquid with respect to the initial situation. Pressure development was measured in the resuscitation balloon during the 5th pulse to minimize slope effects. Pressure was controlled and recorded with an intravenous blood pressure sensor (501669001, Merit OEM, the Netherlands) interlinked to a microcontroller (Arduino Uno, Arduino), see Fig. 4. Raw data were filtered with a High-pass Butterworth filter and a Moving Average filter (Matlab R2016b, MathWorks, Natick, USA).

### Statistical Analysis

GraphPad Prism 5 was used to perform statistical analysis. Results were considered statistically significant at the 5% level. A one-way ANOVA and a Tukey *post hoc* test was used for experiments with different SMA configurations and activation frequencies. A repeated measures ANOVA with a Tukey *post hoc* test was used for experiments with different pulse intervals. Results are reported as mean ± SD. Error bars represent 95% Confidence Interval.

## Results

### Design Configurations

**Figure 5:**
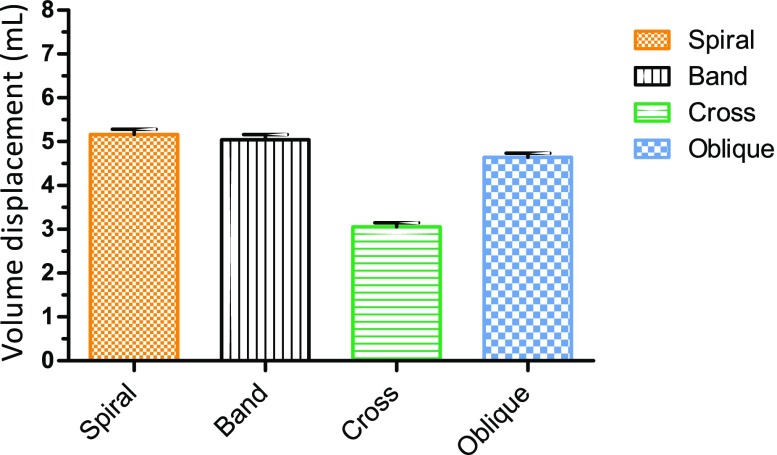
Design of SMA and effect on volume displacement with mean and 95% CI of absolute volume displacement (*p* < 0.0001 for all comparisons, except for spiral vs. band, *p* = 0.08).

SMA wires were applied onto the resuscitation balloon in spiral, band, cross and oblique configurations. Absolute volume displacement differed significantly for peaks 3–7 between configurations, ANOVA(*F*(3,16) = 652, *p* < 0.0001). A Tukey *post hoc* test indicated significant comparisons between all designs (*p* < 0.0001) favoring spiral design, except for spiral vs. band (*p* = 0.08, 95% CI − 0.016 to 0.26), see Fig. 5.

**Figure 6:**
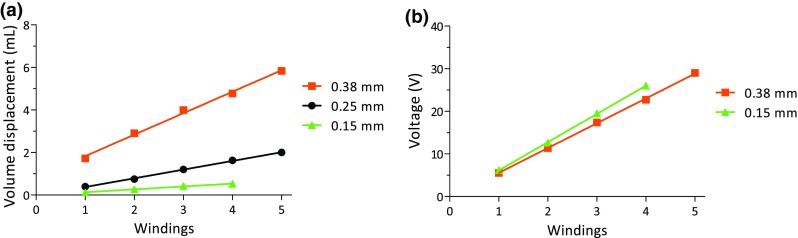
Influence of SMA diameter and windings on (a) volume displacement and (b) power supply. A linear regression line is drawn between data points (*r*^2^ = 0.99 in both graphs).

The influence of wire diameter on volume displacement was studied for 150, 250, and 380 µm SMA wires. Maximum volume displacement increased almost linearly with increasing number of windings around the resuscitation balloon (*r*^2^ = 0.99) in these wires (Fig. 6a). Volume displacement was significantly higher for the 380 µm wire (3.8 mL ± 1.6) as compared to the 250 *µ*m SMA wire (1.2 mL ± 0.6, *p* < 0.05). A similar dependency was observed for the required voltage (Fig. 6b).

**Figure 7:**
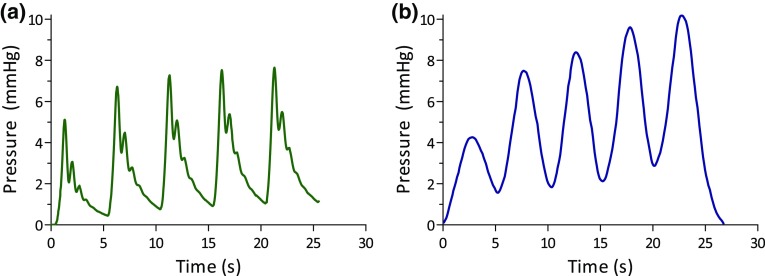
Pressure development with (a) measured volume displacement (mm Hg) and (b) external pressure sensor (mm Hg).

Maximum absolute volume displacement in spiral configuration was 9.0 ± 0.18 cm H_2_O, equivalent to 6.9 ± 0.076 mmHg for pulses 3–5 (Fig. 7a). The calculated pressure in the resuscitation balloon using our pressure sensor was 7.1 ± 0.49 mmHg (Fig. 7b), not statistically significant (*p* = 0.54, 95% CI 5.9 to 8.3). A band shaped configuration with 5 windings resulted in a pressure of 10.3 mmHg at an electric current supply of 2.25 A. A current of 2.85 A in combination with assisting rings to distribute forces across the resuscitation balloon, resulted in maximum volume displacement of 16.4 mL. This corresponds to a pressure build-up of 24.1 mmHg.

### Pulse Width Modulation (PWM) Techniques

**Figure 8:**
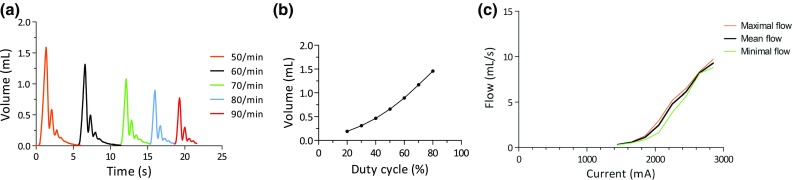
Pulse Width Modulation (PWM) techniques and effect on volume displacement with (a) pulse frequency; (b) duty cycle; (c) electric current on flow (mL/s).

Volume displacement of every third pulse was measured at different frequencies with duty cycle kept constant at 75% and pulse interval at 5 (1/5th of frequency). Maximum displaced volume decreased significantly upon increasing pulse frequency, ANOVA(*F*(4,25) = 400, *p* < 0.0001) (Fig. 8a).

Furthermore, volume displacement reached a constant volume after 3 pulses, regardless of the employed pulse frequency. A Tukey *post hoc* test revealed a significantly higher volume displacement for a frequency of 50/min (1.6 mL ± 0.04, *p* < 0.05) as compared to other frequencies. Both higher duty cycles and higher electric currents increased volume displacement and flow (Figs. 8b and 8c). A duty cycle of 90 or 100% would impair the cooling process and was not tested.

**Figure 9:**
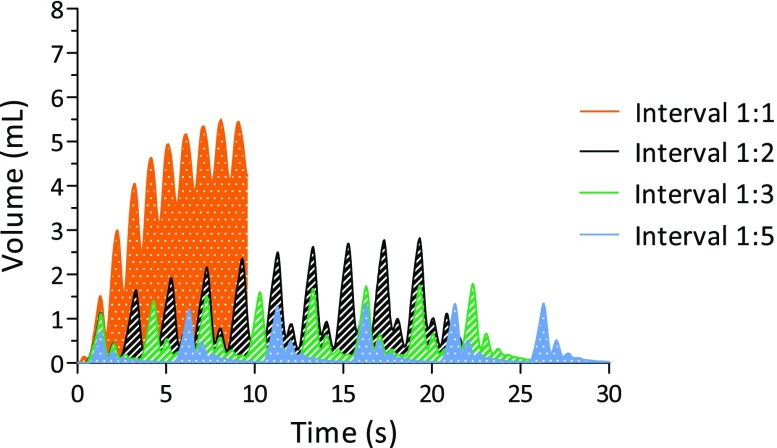
Pulse interval and effect on volume displacement. Volume displacement changes drastically when every cycle (interval of 1) is supported in comparison to other pulse intervals.

Volume displacement changed remarkedly each cycle when supporting every heartbeat at a frequency of 60/min, as compared to other intervals. The SMA was unable to return to its original state within this short cycle time as depicted in Fig. 9. A Tukey *post hoc* correction revealed that absolute volume displacement was statistically significant different for an interval of 1 (2.0 mL ± 0.4) and the intervals 3 (1.4 mL ± 0.2, *p* < 0.01), 4 (1.3 mL ± 0.1, *p* < 0.001) and 5 (1.2 mL ± 0.2, *p* < 0.001). There was no significant difference between interval 1 and 2 (1.8 mL ± 0.4, *p* = 0.35).

## Discussion

In this study we investigated optimal design characteristics of a demonstrator SMA support device for HFrEF patients. A 380 µm SMA Flexinol^®^ wire configured in a spiral oriented shape around a mono-chamber balloon activated with a duty cycle of 80%, a frequency of 50/min gave the highest volume displacement (stroke volume, 6.2 mL). A maximum ejection fraction (EF) of 3.5% was achieved, requiring a power supply of 25 V and 2.85 A for each pulse.

Tozzi *et al*. used a 100 *µ*m diameter SMA nitinol wire with total stimulation of 180 ms, recovery time of 250 ms and contraction rate of 80/min.^23^ They achieved a maximum ejected volume of 330 mL/min, a generated systolic pressure of 75 mmHg, ejection fraction of 12% requiring a power supply of 6 V and 250 mA.^23^ This is comparable to our maximum ejected volume of 310 mL/min. In their experiments, a double-chamber setup was used, with balloon volume of only 50 mL. This is much smaller than patients with HFrEF^22^ making extrapolation of these results into a physiological situation difficult.

### Diameter and Volume Displacement

Volume displacement and required voltage displayed a linear incremental relation in our experiment with the employed number of SMA-wires (windings) for all tested diameters (150, 250 and 380 *µ*m), (Fig. 6a and b). This may suggest that the number of required SMA-wires for a specific volume displacement and the corresponding voltage may be predicted. An EF of 10% for this balloon (= 475 mL) would then require 46 windings, although maximum strain of SMAs might limit this linear behavior.

### Thermal Management

A lower pressure build up was observed in the first contraction cycles (Figs. 7a and 7b). Joule heating started the transformation phase but was not completed yet. After a few cycles temperature increased and the transformation phase was completed.^11^ A linear relationship between electric current increase and volume displacement was observed by Velazquez *et al*.^24^ Indeed, we observed that SMA wires had a higher volume displacement when increasing electric current (Fig. 8c). However, increasing electric currents above the plateau phase as observed in Fig. 8c, may cause overheating and induce stress fatigue. To reduce heating of surrounding tissue, a silicone tube proved to be effective in goat experiments.^21^ Indeed, Shiraishi *et al*. found no major draw backs related to heat production in silicone insulated SMA-wires in goats.^21^ Kalogerakos *et al*. found that insulated wires produced a lower volume displacement than non-insulated wires.^12^ Furthermore, cooling time is slower than heating time and thus urges for interval use such as once in four heart beats.

### Pulse Width Modulation

Heart support during every heartbeat requires rapid compression and expansion of the SMA. This may not be directly attainable, though HF patients may already benefit from partial heart support. Hence the influence of pulse width modulation on volume displacement was investigated. SMA wires that were left to cool down before the following activation step produced substantially higher volume displacements (Fig. 9). In our experiment, pulse intervals of every 3 or every 5 cycles gave higher volume displacements than pulse intervals of every 1 or every 2 heart beats. A second, lower peak (“bounce”) was absent with a pulse interval of 1. Here, next activation occurred faster than cooldown and recovery of the SMA wire.

In this demonstrator study, hourly energy consumption of SMA activation ranged from 5.5 to 11.4 Watt-hour, where an increased energy use resulted in higher ejection fractions. This range is lower than the hourly energy consumption of 23.6 Watt-hour of a HeartMate II assist device^25^ and is comparable to SMA-prototypes^12^ or HeartMate III at 11 Watt–hour.^18^ At body temperature, SMA wires consume less energy than bench temperature due to a reduction in temperature gradient.^10^ This is promising for battery size reduction and therefore reduces frequent change of the (external) battery.

### Limitations of Experimental Setup

A silicon pediatric resuscitation balloon was employed as a static model and acted as a mono-chamber model with a large end-diastolic volume (EDV) of 475 mL. EDV in HFrEF-patients range from 100 to 300 mL, depending on ventricular dilatation.^22^ Thus, this model underestimates effect size for clinical use.

Durability of SMAs for cardiac applications should extend beyond patients life expectancy. In this study, no change in volume displacement was observed after 25 min (Supplemental Fig. 1). Comparable SMAs were tested in a fatigue lifetime test and assured constant stroke after at least 10^6^ cycles.^7^ However, for cardiac applications these experiments should ensure SMA stability over 10^8^ cycles.

Also, afterload is reduced in LVAD therapy where effects of a SMA support device on afterload are still unknown. These effects should be addressed in future experimental setups and should be within physiological systemic aortal pressure.

Finally, energy consumption for achieving an ejection fraction of 3.5% was threefold higher than a pacemaker. This might affect future clinical use when battery life is too short for internal battery use. External battery use comes with an increased risk for driveline infections and impair patients mobility.^16^ An increase of parallel windings can reduce SMA length and resistance, with a reduction in energy consumption to follow.

## Future Perspectives

Future research should focus on evaluation of spiral or oblique shaped configurations around the heart in advanced bench models such as dynamic setups or an *ex vivo* porcine cadaver model. The helical ventricular myocardial band cardiac fiber orientation should be acknowledged in these designs.^13^ This represents a physiologically more realistic model than the proof of principle in an *in vitro* mono-chamber bench model presented here. Alternatively, a double-chamber bench model with incorporated right ventricle can then include biventricular support. This should increase the technology readiness level from 3 (Characteristic Proof-of-Concept) to 4 (Proof-of-Concept in defined models). Furthermore, combining both different afterload scenarios such as hypertension or mitral regurgitation and design configurations in a mathematical model might enhance device development.

Effectivity of SMA-wire application may be objectified with (*ex vivo*) Magnetic Resonance Imaging (MRI) on segmental wall motion abnormalities. Also, a mesh such as the CorCap Cardiac Support Device or other supportive materials may be incorporated around the heart in future designs to guide and support SMA-wires and prevent dilatation of the left ventricle. This would also prevent incising heart tissue with SMA-wires. The resultant exoskeleton then prevents occurrence of dead space between SMA-wires and epicardium, thus giving additional support for effective contraction and improved ejection fraction.

An increased stroke volume of 11.8 mL improve patients New York Heart Association (NYHA) functional class on heart failure symptoms by a class.^12^ Our current setup might improve these symptoms by half a class, which is not clinically relevant yet. Future studies should aim to improve NYHA-class at least by one and preferably by two classes.

## Conclusions

Optimal design characteristics of a SMA support device for HFrEF patients were determined in this demonstrator study. A LVEF increase of 3.5% was demonstrated in a mono-chamber static heart model by activation of SMA-wires. Furthermore, effects of configuration, duty cycle, frequency, pulse intervals and diameter on the activation of SMA-wires and corresponding volume displacement was evaluated.

## Electronic supplementary material

Below is the link to the electronic supplementary material.

Supplementary material 1 (DOCX 310 kb)
