# Supplementary material for: Technical Feasibility and Design of a Shape Memory Alloy Support Device to Increase Ejection Fraction in Patients with Heart Failure
Source: Cardiovasc Eng Technol. 2019 Jan 9;10(1):1–9. doi: 10.1007/s13239-018-00399-7 (PMC6394801; doi:10.1007/s13239-018-00399-7)
Supplement: Supplementary file 1 — Supplementary material 1 (DOCX 310 kb) [file 13239_2018_399_MOESM1_ESM.docx]

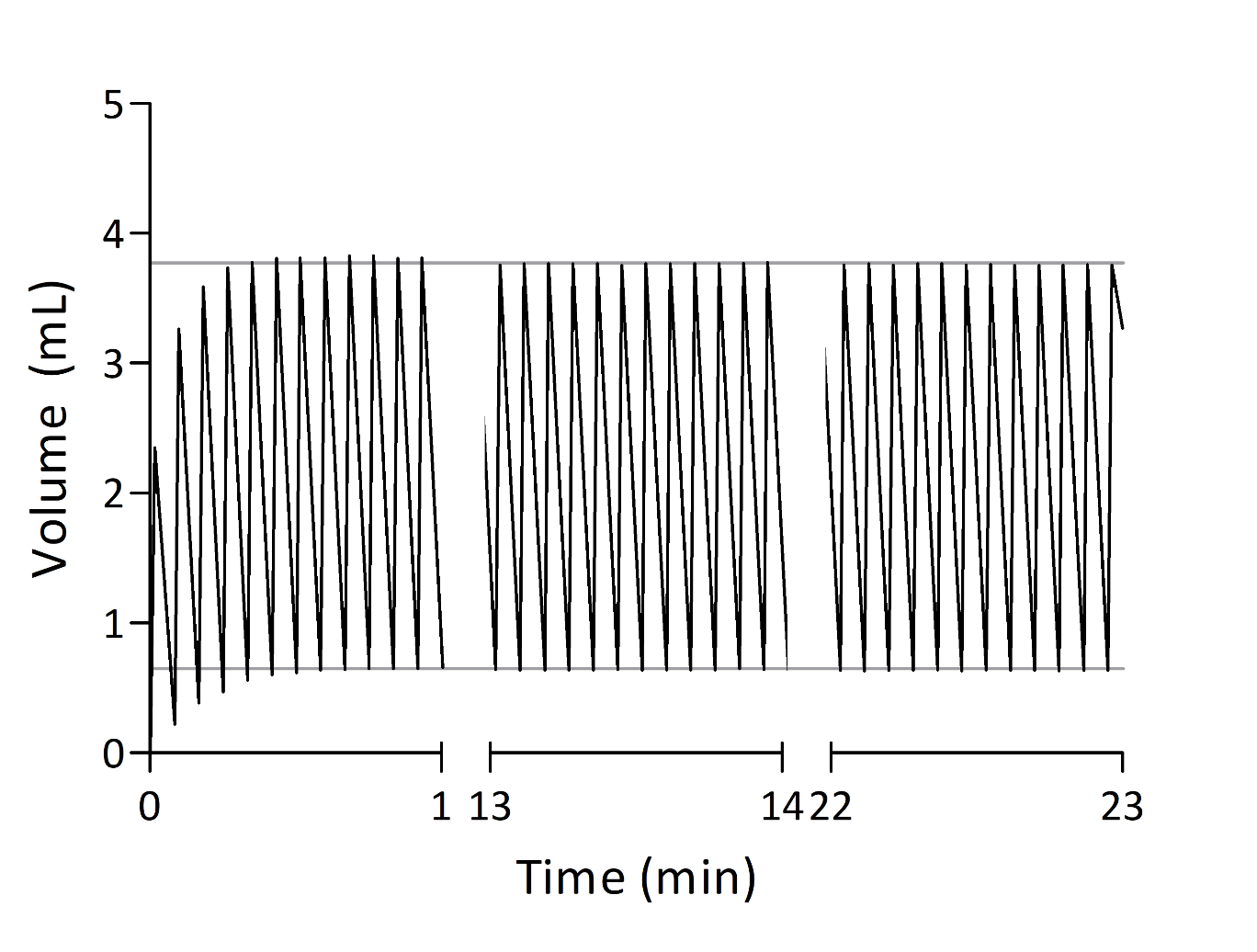


**Supplementary Fig. 1** Volume displacement of two 380 μm SMA-wires in time. A representative selection of 1^st^, 13^th^ and 22^nd^ minute is shown where volume displacement is stable in time. Horizontal grey lines represent mean of highest and lowest measurement.
